# Ammonia in Bronchial and Lung Troubles

**Published:** 1877-03-20

**Authors:** A. H. Patton

**Affiliations:** Vincennes, Ind.


### AMMONIA IN BRONCHIAL AND LUNG TROUBLES.

Dr. A. H. Patton, thus writes to the *Virginia Medical Monthly* :

*Dear Doctor* :—* * * The article in your January number, 1877, by Dr. Wharton (*How to cure a Bad Cold*), although short, is valuable, as it teaches a fact in regard to carbonate of ammonia in antagonizing hyperinosis that ought to be known and appreciated by all physicians.

I claim some originality in the use of ammonia and other alkalies in the treatment of pneumonia, acute bronchitis, and common catarrhal affections. I have been employing alkalies of various kinds in catarrhal affections since 1846, and carbonate of ammonia as the leading remedial agent in pneumonia since 1862—the use of the medicines in these cases being forced upon me by the exigencies of war. While the First Mississippi Regiment was encamped on Brazos Island, in 1846, though the weather was hot enough, the men suffered from "bad colds," and there being no medical stores in reach, I improvised a cough syrup that acted like a charm. New Orleans molasses and bicarbonate of soda apparently constituted a rather unscientific formula which gained me favorable notice at headquarters, and lead to my elevation from the position of a private to the charge of a hospital at Matamoras. Since then I have seldom failed to employ the alkaline treatment in the acute stage of colds.

The ammonia treatment of pneumonitis was forced upon me when I was Brigade Surgeon of Maxey's Brigade, while encamped at Port Hudson during the late war. Thirteen cases of pneumonia were presented for treatment, and the only medicine at my command that was likely to prove beneficial, was carbonate of ammonia. I was astonished to find that the improvement in all the cases was very marked and decided from large doses of the ammonia—from five to ten grains every two hours, in a quarter of a glass of water. In 1870 I published an article in the *American Journal of Medical Sciences*, on Carbonate of Ammonia in Pneumonitis, in which I advocated its use in the doses mentioned from the first day of treatment until the cure was complete. I supposed this to have been the introduction of a plan of treatment that has since become very general and highly successful.

Of course I am well aware that the ammonia has long been used in the advanced stages of the disease, mainly for its supposed stimulant power. Dr. Flint advocated its use to prevent embolism, but I was the first to advise it in the early stages as a means of overcoming the controlling element of danger in the disease—hyperinosis. I regret that there are still large numbers of physicians who fail to properly esteem the extraordinary powers of this medicine in pneumonia. With the exception of blisters, which I employ in the first stage, I use little else in that formidable disease but carbonate of ammonia Opiates and quinine are sometimes indicated and given ; but calomel, tartar emetic, squills, or any other depressing medicine, are never given in any case of pneumonia that comes under my care.

Truly yours,

A. PATTON, M. D.

*Vincennes, Ind.*

